# Facing tuberculosis during the COVID-19 pandemic: the perspective of those who experienced it

**DOI:** 10.1590/0034-7167-2024-0310

**Published:** 2025-03-14

**Authors:** Thaynara Eloise Baracho de Albuquerque Farias, Hellen Cristina Sthal, Valdízia Mendes e Silva, Aguinaldo José de Araújo, Paula Hino, Roxana Isabel Cardoso Gonzales, Maria Rita Bertolozzi, Tânia Maria Ribeiro Monteiro de Figueiredo

**Affiliations:** I Universidade Estadual da Paraíba. Campina Grande, Paraíba, Brazil; II Universidade Federal de Goiás. Goiânia, Goiás, Brazil; III Universidade Federal do Rio Grande do Norte. Natal, Rio Grande do Norte, Brazil; IV Universidade Federal de São Paulo. São Paulo, São Paulo, Brazil

**Keywords:** Tuberculoses, Pandemic, COVID-19, Health Vulnerability, Public Health, Tuberculosis, Pandemia, COVID-19, Vulnerabilidad en Salud, Salud Pública

## Abstract

**Objectives:**

to understand the repercussions of the COVID-19 pandemic on the management of tuberculosis, from the perspective of individuals who experienced the disease during this period.

**Methods:**

this is a descriptive study with a qualitative approach. Eleven individuals participated in the study, and semi-structured interviews were conducted. The data were processed using the IRAMUTEQ software (*Interface de R pour les Analyses Multidimensionnelles de Textes et de Questionnaires*) and analyzed using content analysis techniques, as proposed by Bardin.

**Results:**

four categories emerged, through which it was possible to identify that the pandemic contributed to generating or exacerbating programmatic and social vulnerabilities, such as the lack of home visits and Directly Observed Treatment, treatment interruption, delays, and errors in diagnosis, as well as stigmatization.

**Final Considerations:**

There is an inferred need to strengthen policies and investments to ensure that tuberculosis control and monitoring actions are maintained, even during public health crises.

## RESUMEN

**Objetivos:**

comprender las repercusiones de la pandemia de COVID-19 en el enfrentamiento de la tuberculosis, desde la perspectiva de las personas que experimentaron la enfermedad durante este periodo.

**Métodos:**

investigación descriptiva con enfoque cualitativo. Participaron 11 individuos en la investigación, y se realizaron entrevistas semiestructuradas. Los datos fueron procesados utilizando el *software* IRAMUTEQ (*Interface de R pour les Analyses Multidimensionnelles de Textes et de Questionnaires*) y analizados mediante la técnica de análisis de contenido propuesta por Bardin.

**Resultados:**

emergieron cuatro categorías a través de las cuales fue posible identificar que la pandemia contribuyó a generar o potenciar vulnerabilidades programáticas y sociales, tales como: la no realización de visitas domiciliarias y del Tratamiento Directamente Observado, la interrupción del tratamiento, retrasos y errores en el diagnóstico, además de la estigmatización.

**Consideraciones Finales:**

se infiere la necesidad de fortalecer las políticas e inversiones para que las acciones de control y seguimiento de la tuberculosis se mantengan, incluso en períodos de crisis sanitarias.

## INTRODUCTION

COVID-19 was responsible for the largest pandemic in recent decades^(1)^. Its consequences went beyond the high morbidity and mortality rates caused by the Sars-Cov-2 virus, significantly impacting the management of pre-existing diseases, such as tuberculosis (TB), which continues to be a major public health challenge worldwide^(2)^.

In 1993, TB was declared a global emergency by the World Health Organization (WHO), establishing disease control as a goal for countries^(3)^. Since then, global efforts have intensified to address this challenge. In 2015, the seventeen Sustainable Development Goals (SDGs) were defined, and the commitment to eliminate TB by 2030 was incorporated into Target 3.3 of the third SDG - Health and Well-being^(2)^.

However, achieving this goal became even more challenging with the COVID-19 pandemic, which had a significant impact on healthcare services, affecting TB management activities, such as a decrease in TB case notifications and an increase in the number of deaths caused by the disease^(4)^.

The necessary measures to contain the rapid spread of COVID-19, such as social distancing and the recommendation to reduce the frequency of healthcare visits, posed additional challenges for those dealing with a serious illness like TB, making them “invisible” as financial resources, services, and professionals were redirected to people infected with COVID-19^(5)^.

Moreover, it is important to highlight that TB, despite being one of humanity’s oldest diseases, bears the burden of being a socially stigmatized illness. It not only affects the physical body but also disrupts daily life, impacting family, work, social relationships, and even mental health^(6)^. The pandemic exacerbated these vulnerabilities, creating greater fear and concern by making individuals with TB more susceptible to adverse outcomes, such as death, in the event of COVID-19 infection^(4,7)^.

In this context, it is essential to understand how people with TB managed to cope with the disease in an adverse scenario marked by uncertainties, which, in Brazil, was further aggravated by the spread of false information, often conflicting with global health recommendations^(8)^.

Thus, it is important to understand, from the perspective of those who experienced TB during the pandemic, the aspects that generated or intensified vulnerabilities, which can be analyzed from three interdependent dimensions: individual, which involves personal characteristics, behaviors, and knowledge about the disease; social, related to the social context, access to and quality of information; and programmatic, involving institutions, especially healthcare services, policies, programs, and services. These three dimensions provide a framework for analyzing and understanding a unique reality, such as TB illness and management, which is experienced in a singular and subjective way by each individual^(9)^.

Therefore, understanding the perceptions of people who experienced pulmonary TB treatment, recognizing how the pandemic interfered with their disease management in terms of diagnosis, treatment adherence, access to services, and the assistance provided by healthcare professionals, is crucial for gathering experiences and knowledge in the fight against TB. Additionally, it contributes to strengthening strategies to achieve the global targets set in the End TB Strategy and the SDGs^(2,10)^.

## OBJECTIVES

To understand the repercussions of the COVID-19 pandemic on the management of TB from the perspective of individuals who experienced the disease during this period.

## METHODS

### Ethical considerations

The study adhered to the ethical guidelines of Resolution No. 466/2012 of the Brazilian National Health Council, with approval from the Ethics Committee of the State University of Paraíba. Participants were informed about the study, signed the Informed Consent Form (ICF), and the Voice Recording Authorization Form, with anonymity guaranteed for all interviewees.

### Theoretical-methodological framework

The study was based on the concept of “vulnerability”, as described by Ayres and collaborators, which is understood through three dimensions: the individual dimension, which depends on a person’s lifestyle and can contribute to increasing or reducing vulnerability. This is intrinsically linked to personal characteristics, information, knowledge about the disease, as well as interests, beliefs, values, attitudes, behaviors, and emotional relationships^(11,12)^.

The social dimension, on the other hand, relates to access to and the quality of information, focusing on people’s values and interests, access to communication channels, availability of material resources, social norms, cultural references, gender relations, race, employment, social well-being, stigma, and discrimination^(11,12)^.

Finally, the programmatic dimension involves institutions, particularly healthcare services, including programs, policies, actions, access to services, quality of services, comprehensive care by multidisciplinary teams, interdisciplinary approaches, integration of prevention, promotion, and care, as well as the training and commitment of healthcare professionals^(11,12)^.

### Study design

This is an analytical study with a qualitative approach, following the recommendations of the Consolidated Criteria for Reporting Qualitative Research (COREQ).

### Study setting

The study was conducted in one of the largest municipalities in the interior of Northeast Brazil, with an estimated population of 419,379 inhabitants^(13)^. During the data collection period, the municipal TB Control Program consisted of 80 Basic Health Units, distributed across 7 health districts, with 105 Family Health Strategy teams, 1 Reference Clinic for TB and Leprosy, 6 health centers, 2 polyclinics, and 1 street outreach team^(14)^.

### Data source

The population consisted of all individuals diagnosed with the pulmonary form of TB and classified as new cases. Inclusion criteria were being 18 years or older and having undergone treatment in 2020 or 2021 at a Family Health Strategy unit in the urban area or the Reference Clinic. Cases diagnosed in individuals deprived of liberty and those with incomplete or incorrect addresses were excluded.

The sample was obtained through convenience sampling and consisted of 11 individuals, determined by the theoretical saturation criterion, which occurs when no new elements emerge from the collected information, leading to theoretical consolidation due to repetition^(15)^.

### Data Collection and Organization

To access the participants, initial contact was made with the municipality’s epidemiological surveillance coordination, which provided a list of individuals affected by TB, registered in the National System of Notifiable Diseases (SINAN in Portuguese) between 2020 and 2021. Data collection took place in January and February 2024. Additionally, no participants refused to take part in the study.

The interviews were conducted by a nurse researcher, a graduate student, and two undergraduate nursing students from a public higher education institution, members of the research group “Evaluation of Health Services” (UEPB/CNPq), who were trained by the research coordinator. Participants were approached in person, and the interviews were conducted in their homes.

For data collection, a semi-structured interview guide was used, developed by the researchers, containing questions about sociodemographic characteristics and the following guiding questions: “Describe how your TB diagnosis and treatment occurred during the COVID-19 pandemic”. “Talk about your access to health services for TB treatment during the pandemic”. “Describe the care provided by health professionals during the pandemic”. The guide was previously tested on an individual who underwent TB treatment, who was not part of the study sample, to assess the clarity of the guiding questions. The interviews were audio-recorded and had an average duration of nine minutes. Immediately after the interviews were conducted, they were fully transcribed, with feedback provided to those who expressed interest in receiving a copy for potential corrections.

### Data Analysis

The participants’ responses were organized into a textual corpus and then processed using the software IRaMuTeQ (*Interface de R pour les Analyses Multidimensionnelles de Textes et de Questionnaires*), a tool that enables various types of textual data analysis, including Descending Hierarchical Classification (DHC), as proposed by Reinert ^(16)^, which allows for the identification of classes of text segments with similar vocabulary among themselves and vocabulary different from other text segments^(17)^.

For this study, we used the Content Analysis technique proposed by Bardin^(18)^, through thematic categorization, encompassing three stages: pre-analysis, in which the interview transcripts underwent floating reading; followed by an exhaustive reading of the material, selecting the recording units. Finally, the recording units were organized into categories and interpreted according to the theoretical framework and the relevant literature. As a result, four thematic categories emerged: the repercussions of the COVID-19 pandemic on access to health services; care for people with TB during the pandemic; implications of the pandemic for TB diagnosis; and implications of the pandemic for TB treatment adherence.

## RESULTS

Of the 11 individuals who participated in the study, the majority were female (63.6%), over 30 years old (72.7%), identified as white (54.6%), had completed higher education (45.4%), and held formal employment (45.4%), with an income between one and two minimum wages (72.8%). Regarding TB care, most participants were followed by the UBS (72.2%), and all (100%) self-administered their treatment.

The textual corpus, composed of participants’ responses about their experience with TB during the pandemic-covering diagnosis, treatment, access to health services, and the assistance provided by healthcare professionals-presented 5,908 word occurrences and 1,156 distinct forms, with a text utilization rate of 81.18%, which is considered satisfactory for DHC analysis.

**Figure 1:**
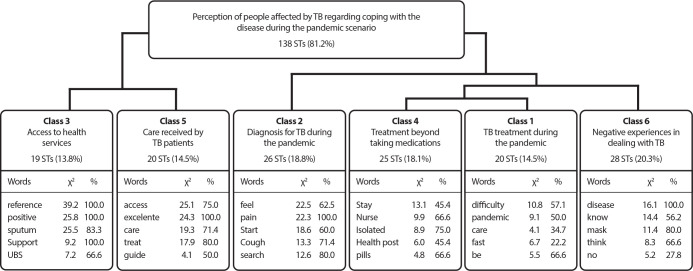
Diagram of the classes that make up the dendrogram of the textual corpus regarding the perception of people affected by tuberculosis, Campina Grande, Paraíba, Brazil

The lexicographic analysis of the corpus, using DHC, grouped the texts into two divisions and six classes, as shown in the diagram that illustrates the dendrogram produced by the Iramuteq analysis (Figure 1). The words that stood out in each class are also highlighted, selected from those with a chi-square (x^2^) value equal to or greater than 3.84, followed by the respective percentages of text segments containing the word in each class.

*ST - Number of text segments; X^2^ - chi-squared association of the word with the class; % - percentage of segments containing the word in the class.*

Based on the classes and text segments highlighted by the DHC, thematic categories were constructed, supported by the Content Analysis technique proposed by Bardin (2016), as follows:

### Category I - Repercussions of the COVID-19 pandemic on access to health services

Category I emerged from Class 3, which grouped individuals’ perceptions of access to health services during the pandemic. It was noted that family support was essential for people with TB to avoid Sars-Cov-2 contamination when accessing health services to continue their TB treatment, such as picking up medications at the UBS and delivering sputum samples for monthly monitoring at the TB Reference Clinic.

[...] *I stayed home, and a family member would take the sputum test for me due to the pandemic, so I wouldn’t risk getting COVID in addition to TB. This support network was essential*. (E9)

The TB follow-up provided by Primary Health Care (PHC), which was already in place before the pandemic, was crucial for maintaining treatment, as the proximity of the UBS to residences and the strong relationship between professionals and the community made a difference.

*Access to health services during the pandemic wasn’t difficult. I went to the health center near my house, and my father picked up the medication at the clinic.* (E3)

*I had all the family support I needed. Whenever I needed it, someone would pick up the medications* […]. (E9)

### Category II - Care for people with tuberculosis during the pandemic

The second category emerged from Class 5, which included statements describing the healthcare received as good and welcoming, as seen in the following excerpts:

*The care provided by healthcare professionals during the TB treatment was good.* (E3)

[...] *the staff treated me well; they were very welcoming.* (E1)

However, the measures imposed to contain the pandemic, such as social distancing, may have compromised the care provided by healthcare professionals, as they stopped performing essential activities for TB follow-up, as illustrated in the following excerpts:

*To be honest, the care provided by the healthcare professionals, they’re not even coming here. The UBS is near my house, but they haven’t come. They haven’t visited me yet.* (E4)

*I didn’t have follow-up from the healthcare professionals. I thought the people from the health unit would be visiting me.* (E9)

Two interviewees reported interruptions in their treatment, which led to complications such as worsening clinical conditions.

[...] *the only thing that happened was a lack of medication for a week at the clinic because the staff were getting sick and being put on leave, so there was this setback.* (E10)

*My treatment was interrupted, which wasn’t supposed to happen. My concern was that they lost my file at a health unit.* (E5)

### Category III - Implications of the pandemic in seeking a tuberculosis diagnosis

The third category emerged from Class 2. In most reports, the diagnosis was made in a timely manner. However, for some interviewees, it took more than 30 days to conclude the diagnosis. Additionally, they had to access more than one healthcare service, further increasing the risk of contracting COVID-19.

*It had already been quite a while, about 40 days or so, and I kept going to the clinic.* (E01)

*I kept going to the pulmonologist almost all year, being treated as an asthmatic, and time was wasted. Then in 2020, I went to another doctor. When he looked at me, he said, “Go take this TB test”.* (E7)

*The bad thing was being sick and having to go to places where I could catch COVID, exposing myself more and more.* (E8)

There were also reports of misdiagnoses and delays in diagnosis. As a result, there were setbacks in distinguishing TB from other diseases, such as the flu, COVID-19, pneumonia, and cancer.

*The doctor said I had early-stage anxiety. But I didn’t. I told her I wasn’t anxious, that I was having a lot of pain in my ribs, and that I could have had pleural effusion. She said, ‘No, it’s just a little flu,’ and she prescribed me medication.* (E08)

### Category IV - Impact of the pandemic on tuberculosis treatment

This category emerged from Classes 4, 1, and 6. In Class 4, some statements highlighted the role of the nurse working at the UBS as a key figure in establishing a strong user-service relationship, capable of encouraging adherence to TB treatment and achieving a cure, as observed in the following excerpt:

*The nurse at the clinic was very good. She had a strong spirituality. I really liked the way she was, and at the end of the treatment, I thanked her because she had very high self-esteem.* (E01)

Other interviewees mentioned receiving guidance to prevent TB transmission through the separation of personal items, a scientifically incorrect practice that can lead to discriminatory behavior:

*All my things were kept separate: the plate, the cup.* (E10)

*I was told that for 15 days, I had to be in complete isolation, keeping everything separate at home-utensils and all.* (E1)

In Class 1, the impact experienced by individuals affected by TB in the midst of a world plagued by uncertainties was highlighted. This led to an emphasis on care practices to prevent severe complications, as demonstrated in the following statements:

*Even my family was isolated from me. I live upstairs, so no one had contact with me.* (E10)

*The treatment during the pandemic was much more careful than it had been before* [...] *because the pandemic came to scare us.* (E9)

*First, the biggest initial challenge was wearing a mask. I had to be at home with a mask on.* (E9)

For some interviewees, the social isolation imposed by the pandemic was a facilitating factor in dealing with the disease, as it was a measure imposed on the general population, contributing to greater discretion:

*It ended up being an ally in the sense that I wasn’t alone, isolated. It wasn’t just me who had to isolate; many people, everyone had to go into quarantine and rest, and that ended up making things better.* (E9)

Class 6, which also forms part of this category, revealed that the pandemic may have contributed to an increase in stigmatization, as observed in the following statement:

*Is it contagious? With all this perspective, this prejudice. The stigma that exists* [...] *people start asking questions, with that prejudice, that perception people have that TB is a poor person’s disease, that it’s something you get when you’re less fortunate. All of that. A disease of poor hygiene, and I didn’t have any of that, my reality was different. People would ask me: where did you catch this?* (E9)

Negative experiences were highlighted, caused by the worsening of TB, such as hospitalization and sequelae, which may have been triggered by delays in TB detection and, consequently, the late start of treatment, as shown in the following excerpts:

*I had a lot of secretion. I had two bronchoscopies, which is a lung wash. The doctor thought it worked, but it didn’t! He said: there are nodules. He thought they were malignant, but they weren’t, and he removed the lower left lobe a year ago.* (E7)

*I spent twelve days in the hospital. Of those twelve days, four were in the ICU, and the doctor even wanted to intubate me. I wasn’t intubated only because my aunt didn’t allow it.* (E8)

## DISCUSSION

The content analysis of the interviews allowed us to understand the factors or situations that either facilitated or hindered, in accordance with the dimensions of vulnerability, the diagnosis, adherence to treatment, access to services, and the care provided by healthcare professionals.

The sociodemographic characteristics of most participants did not suggest individual vulnerabilities, unlike findings in another study, which highlighted factors such as low education levels, alcohol and drug use, psychological disorders, and lack of family support as elements that could interfere with adherence to TB treatment^(19)^.

The family’s role as a co-responsible party in the treatment, as highlighted in the first category, was essential in minimizing the risk of TB patients being exposed to COVID-19 when accessing healthcare services. Family support is crucial, as it helps individuals overcome difficulties and contributes to treatment success^(20,21)^.

Another factor that may have contributed to the lack of challenges in accessing services during the pandemic was the follow-up and treatment of TB by PHC, which, according to most accounts, did not present access barriers. This may have been due to the geographic proximity of participants’ homes to the PHC centers^(22)^.

Additionally, the care provided to TB patients, discussed in the second category, requires an approach focused on building a strong relationship between patients and healthcare teams and services. This is critical for managing the disease, as it allows for the identification of individual needs and the understanding of vulnerabilities that may affect outcomes, enabling the development of specific care strategies with the participation of other services^(23)^.

In this sense, the work of healthcare teams should be guided by actions that promote treatment adherence, such as providing support, active listening, home visits (HV), Directly Observed Treatment (DOT), and health education activities that help overcome stigma and discrimination, among others^(24)^.

The pandemic context, with the necessary measures to contain the spread of COVID-19, such as social distancing, may have compromised TB control and follow-up activities^(24)^, as emphasized by the participants in this study. These issues represent programmatic vulnerabilities, reflecting deficiencies in healthcare systems^(23)^, such as the lack of HV and DOT, given that all participants self-administered their treatment.

HV is one of the recommendations from the Ministry of Health to strengthen the relationship between services and patients, as well as ensure treatment adherence. It is recommended that HV be offered whenever possible to all patients, especially at the start of treatment, as it provides an opportunity to understand the social context in which they live^(3)^.

DOT is also a fundamental, humanized, and committed tool for monitoring treatment. Its implementation allows professionals to further engage with patients’ social contexts^(23)^. As such, DOT is intended for all individuals diagnosed with TB and involves supervising medication intake three to five times per week to reduce treatment interruptions^(3)^.

The COVID-19 pandemic intensified challenges but also revealed vulnerabilities that should no longer exist, particularly within PHC, as TB treatment is a priority according to the Basic Healthcare Policy^(6)^.

In this context, it is important to mention that the National Plan to End TB supports the development of strategies that utilize, for example, digital technologies^(2,10)^. Similarly, international and national guidelines for TB management and control during the pandemic emphasized the need to strengthen relationships with TB patients, even remotely, through phone calls or video calls. However, this approach was not reported by the interviewees, suggesting that such efforts did not occur^(7,25)^.

A study conducted in the state of Paraíba found that there was no funding to facilitate phone calls, and most PHC units did not have phones or mobile devices. As a result, routine PHC activities were halted during the pandemic, highlighting the need for increased investment to ensure TB control efforts and other priority disease programs, even during adverse periods^(26)^.

The reported interruption of TB treatment also demonstrates that the pandemic led to an increase in treatment follow-up losses due to the disorganization or restructuring of services, the reallocation of material and human resources, and the recommendation to reduce the frequency of appointments because of COVID-19. This emphasizes programmatic vulnerability^(27)^.

These vulnerabilities were also observed in a retrospective cohort study conducted in Brazil during 2020 and 2021, which showed that the proportion of follow-up loss increased during the pandemic, reaching approximately 19%, three times higher than what is recommended by the WHO^(3,27)^.

Regarding TB diagnosis during the COVID-19 pandemic, in the third category, it was found that one case experienced a delay of more than forty days and another a delay of up to a year in obtaining a TB diagnosis, although most interviewees were diagnosed in a timely manner. There were also cases of misdiagnosis, further contributing to the spread of the disease in society and indicating programmatic vulnerabilities.

People diagnosed with TB, due to their pulmonary impairment, are part of the risk group for COVID-19 and may present similar symptoms between the two diseases, such as fever and cough. For this reason, they require special attention, focusing on differential diagnosis and early detection^(28)^.

The delay in diagnosis is possibly related to the impact of the pandemic, which led to a decline in TB diagnoses worldwide. According to the Epidemiological Bulletin^(11)^, in Paraíba, 1,144 new cases were reported in 2020, with a reduction in detection compared to the previous year. This demonstrates that services were unable to maintain TB surveillance and control activities during the pandemic^(29)^, which may have contributed to the disease’s spread in society. Therefore, early diagnosis and the immediate start of treatment are crucial TB control measures^(4)^.

In the fourth category, which addresses the impact of the pandemic on TB treatment, three participants (E4, E9, and E7) reported worsening symptoms, leading to hospitalization. Among them, E7, due to delayed diagnosis, had to undergo a pulmonary lobectomy (removal of the lower left lobe) and now lives with this sequela. These findings confirm that, despite being a curable disease, programmatic vulnerabilities can lead to unfavorable outcomes and highlight the need for better preparation of healthcare professionals for early TB detection and diagnosis^(23)^.

Additionally, this category emphasized the support provided by professionals, particularly nurses. Other studies show that nursing plays a key role in managing and controlling the disease, especially in PHC, fostering a mutual relationship between professionals and patients and enabling the recognition of health needs. Through this relationship, trust and autonomy are established, helping individuals with TB overcome fears, concerns, and stigmas related to the disease and its treatment^(26,30)^.

However, it is important to note that incorrect guidance about TB transmission-such as recommending the separation of utensils, cups, and plates-reveals programmatic vulnerabilities. It is well known that the transmission of *Mycobacterium tuberculosis* occurs via airborne particles released through talking, coughing, or sneezing, which remain suspended in the air^(4)^.

Thus, the dissemination of separatist information like this should not come from healthcare professionals, who should provide guidance from the initial suspicion of the disease through to its cure. This guidance should include essential information such as defining TB, prevention methods, the importance of contact tracing, treatment duration, adverse reactions, and the need for treatment adherence to achieve a cure^(20,28)^. Therefore, it is necessary to provide continuous education to these professionals to improve their ability to support TB patients.

In addition to facing TB, patients also had to contend with the COVID-19 pandemic, which heightened the need for respiratory hygiene measures, hand sanitation, and the use of face masks^(31)^ to prevent the spread of the new coronavirus and protect against potential coinfection. These factors may have posed an additional challenge for those already dealing with a serious pulmonary disease like TB. One interviewee reported that wearing a mask was particularly challenging due to difficulty breathing.

Although these measures caused changes in people’s behavioral and emotional aspects, one interviewee saw social isolation as an ally in the fight against TB, since everyone was in quarantine. This meant there was no need to explain the disease, which helped avoid the stigma that still affects those dealing with TB.

Prejudiced conceptions about TB persist today, even though it is a curable disease, further reinforcing stigmatization. As one interviewee reported, TB illness is still associated with reckless behavior and poor hygiene. Prejudice often arises from misguided beliefs about how the disease is transmitted, beliefs that linger in the social imagination, contributing to social vulnerability^(6,20)^.

In this context, it is necessary to create spaces for discussion with the public about TB, particularly regarding its transmission and prevention, so that changes in these perceptions can occur, helping to demystify and raise awareness. In this way, individuals affected by TB can feel welcomed and reintegrated into society.

### Study limitations

The limitations of this study include difficulties in accessing the SINAN database, the small sample size, and incomplete or incorrectly filled data, particularly regarding addresses, which made conducting interviews more challenging.

### Contributions to Public Health

This study provides insights from the perspective of people with TB on how the disease was treated during the COVID-19 pandemic. It highlights the need to strengthen strategies that ensure the management of neglected diseases, such as TB, in the context of a public health emergency.

## FINAL CONSIDERATIONS

The findings reveal that the pandemic contributed to creating or intensifying programmatic and social vulnerabilities that impacted TB management. These vulnerabilities included the lack of HV and DOT, treatment interruptions, delays, and diagnostic errors, as well as the persistence of stigma.

Conversely, family support, TB treatment follow-up by PHC, supportive care, the nurse’s role in enhancing treatment adherence, and social isolation-which helped reduce prejudice-were essential factors in achieving a TB cure in the adverse pandemic scenario.

The findings underscore the importance and urgency of expanding TB control and follow-up efforts to meet the SDGs and eliminate TB as a public health issue. Therefore, it is necessary to promote continuous health education on disease management and control for healthcare professionals, provide public health education to encourage community engagement in the fight against TB, and strengthen policies and investments to ensure the sustainability of TB control measures, even during public health crises.
