# Supplementary material for: Facing tuberculosis during the COVID-19 pandemic: the perspective of those who experienced it
Source: Rev Bras Enferm. 2025 Mar 14;78(Suppl 1):e20240310. doi: 10.1590/0034-7167-2024-0310 (PMC11913131; doi:10.1590/0034-7167-2024-0310)
Supplement: Translation

# Translation: Enfrentamento da tuberculose durante a pandemia de COVID-19: o olhar das pessoas que a vivenciaram

**DOI:** 10.1590/0034-7167-2024-0310pt

**Authors:** Thaynara Eloise Baracho de Albuquerque Farias, Hellen Cristina Sthal, Valdízia Mendes e Silva, Aguinaldo José de Araújo, Paula Hino, Roxana Isabel Cardoso Gonzales, Maria Rita Bertolozzi, Tânia Maria Ribeiro Monteiro de Figueiredo

**Affiliations:** I Universidade Estadual da Paraíba. Campina Grande, Paraíba, Brasil; II Universidade Federal de Goiás. Goiânia, Goiás, Brasil; III Universidade Federal do Rio Grande do Norte. Natal, Rio Grande do Norte, Brasil; IV Universidade Federal de São Paulo. São Paulo, São Paulo, Brasil

**Keywords:** Tuberculose, Pandemia, COVID-19, Vulnerabilidade em Saúde, Saúde Pública

## Abstract

**Objetivos:**

compreender as repercussões da pandemia de COVID-19 no enfrentamento da tuberculose, a partir do olhar das pessoas que vivenciaram a doença nesse período.

**Métodos:**

pesquisa descritiva, com abordagem qualitativa. Participaram da pesquisa 11 indivíduos, e foram realizadas entrevistas semiestruturadas. Os dados foram processados pelo *software* IRAMUTEQ (*Interface de R pour les Analyses Multidimensionnelles de Textes et de Questionnaires*) e analisados por meio da técnica de análise de conteúdo, proposta por Bardin.

**Resultados:**

emergiram quatro categorias, através das quais foi possível identificar que a pandemia contribuiu para gerar ou potencializar vulnerabilidades programáticas e sociais, tais como: a não realização de visitas domiciliares e do Tratamento Diretamente Observado, interrupção do tratamento, atraso e equívocos no diagnóstico, além da estigmatização.

**Considerações Finais:**

infere-se a necessidade de fortalecer políticas e investimentos para que as ações de controle e acompanhamento da tuberculose sejam mantidas, mesmo em períodos de crises sanitárias.

## INTRODUÇÃO

A COVID-19 foi responsável pela maior pandemia das últimas décadas^(1)^. Suas consequências foram além dos elevados índices de morbimortalidade causados pelo vírus Sars-Cov-2, impactando significativamente o enfrentamento de doenças já existentes, como a tuberculose (TB), que ainda persiste como um grande desafio à saúde pública mundial^(2)^.

Em 1993, a TB foi declarada pela Organização Mundial da Saúde (OMS) como uma emergência mundial, estabelecendo como meta para os países o controle da doença^(3)^. Desde então, os esforços globais têm se intensificado para o enfrentamento desse desafio. No ano de 2015, foram definidos os dezessete Objetivos de Desenvolvimento Sustentável (ODS), e o compromisso de eliminar a TB até 2030 foi incorporado na meta 3.3 do terceiro ODS - Saúde e bem-estar^(2)^.

No entanto, o alcance dessa meta tornou-se ainda mais desafiador com a pandemia de COVID-19, que causou grande impacto na prestação de serviços de saúde, trazendo consequências para as ações de manejo da TB, como a diminuição das notificações de casos de TB e o aumento do número de mortes pela doença^(4)^.

As medidas necessárias para conter a rápida disseminação da COVID-19, como o distanciamento social e a própria recomendação de reduzir a frequência dos atendimentos, desafiaram ainda mais aqueles que estavam enfrentando uma doença grave como a TB, tornando-os invisíveis, à medida que recursos financeiros, serviços e profissionais foram destinados às pessoas infectadas pela COVID-19^(5)^.

Além disso, é importante destacar que a TB, apesar de ser uma das doenças mais antigas da humanidade, carrega consigo o fardo de ser uma doença social, ainda estigmatizada, que não atinge apenas o corpo físico, mas também compromete o cotidiano, afetando as relações familiares, laborais, sociais e o próprio psicológico^(6)^. A pandemia acentuou essas fragilidades, gerando maiores medos e preocupações ao tornar os indivíduos com TB mais vulneráveis a desfechos desfavoráveis, como a morte, em caso de contaminação pela COVID-19^(4,7)^.

Nesse sentido, torna-se fundamental entender como as pessoas com TB conseguiram enfrentar a doença em meio a um cenário adverso, marcado por incertezas, que, no território nacional, foi ainda mais agravado pela disseminação de informações falsas e, muitas vezes, divergentes das recomendações sanitárias mundiais^(8)^.

Assim, destaca-se a importância de compreender, a partir da experiência das pessoas que vivenciaram a TB no período pandêmico, aspectos que foram capazes de gerar ou potencializar vulnerabilidades, os quais podem ser analisados a partir de três ângulos ou dimensões interdependentes: individuais, que envolvem características pessoais, comportamentos e conhecimento sobre a doença; sociais, que se relacionam ao contexto social, ao acesso e à qualidade da informação; e programáticas, que envolvem instituições, principalmente as de saúde, políticas, programas e serviços. Essas três dimensões são capazes de fornecer subsídios para a análise e compreensão de uma realidade única, como o adoecimento e enfrentamento da TB, que é vivenciada de forma singular e subjetiva por cada indivíduo^(9)^.

Portanto, conhecer as percepções de pessoas que vivenciaram o tratamento da TB pulmonar, reconhecendo de que maneira a pandemia interferiu no seu enfrentamento, no que se refere ao diagnóstico, à adesão ao tratamento, ao acesso aos serviços e à assistência prestada pelos profissionais de saúde, é fundamental para somar experiências e conhecimentos em prol do controle da TB, além de contribuir para o fortalecimento de estratégias para alcançar as metas globais pactuadas na Estratégia pelo Fim da TB (*End TB Strategy*) e nos ODS^(2,10)^.

## OBJETIVOS

Compreender as repercussões da pandemia de COVID-19 no enfrentamento da tuberculose, a partir do olhar das pessoas que vivenciaram a doença nesse período.

## MÉTODOS

### Aspectos éticos

A pesquisa atendeu às recomendações éticas da Resolução nº 466/2012, do Conselho Nacional de Saúde, com aprovação do Comitê de Ética da Universidade Estadual da Paraíba. Os participantes foram esclarecidos quanto à pesquisa, assinando o Termo de Consentimento Livre e Esclarecido (TCLE) e o Termo de Autorização para Gravação de Voz, assegurando-se o anonimato dos depoentes.

### Referencial teórico-metodológico

A pesquisa apoiou-se no conceito de “vulnerabilidade”, descrito por Ayres e colaboradores, que partem da compreensão da vulnerabilidade em três dimensões: a individual, que depende do modo de viver das pessoas, podendo contribuir para o aumento ou proteção da vulnerabilidade. Dessa forma, está intrinsecamente ligada às características pessoais, informações e conhecimentos sobre a doença, bem como interesses, crenças, valores, atitudes, comportamentos e relações afetivas ^(11,12)^. A dimensão social, por sua vez, relaciona-se ao acesso e à qualidade da informação, com foco nos valores e interesses das pessoas, no acesso a meios de comunicação, na disponibilidade de recursos materiais, nas normas sociais, referências culturais, relações de gênero, raciais, emprego, bem-estar social, estigma e discriminação^(11,12)^.

Por fim, considera-se a dimensão programática, que envolve instituições, principalmente as de saúde, incluindo programas, políticas, ações, acesso aos serviços, qualidade dos serviços, integralidade da atenção pelas equipes multidisciplinares, enfoque interdisciplinar, integração entre prevenção, promoção e assistência, além do preparo e compromisso dos profissionais de saúde^(11,12)^.

### Tipo de estudo

Pesquisa analítica, com abordagem qualitativa, norteada pelas recomendações do *Consolidated Criteria for Reporting Qualitative Research* (COREQ).

### Cenário do estudo

O estudo foi desenvolvido em um dos maiores municípios do interior do Nordeste Brasileiro, com população estimada de 419.379 habitantes^(13)^. O Programa de Controle da TB municipal, no período da coleta de dados, era composto por 80 Unidades Básicas de Saúde, distribuídas em 7 distritos sanitários, onde atuavam 105 equipes da Estratégia de Saúde da Família, 1 Ambulatório de Referência em TB e Hanseníase, 6 centros de saúde, 2 policlínicas e 1 equipe de consultório de rua^(14)^.

### Fonte de dados

A população foi composta por todas as pessoas diagnosticadas com a forma pulmonar da TB e classificadas como casos novos. Adotaram-se como critérios de inclusão: idade igual ou superior a 18 anos e ter realizado o tratamento nos anos de 2020 e 2021, em uma unidade da ESF da zona urbana ou no Ambulatório de Referência. Foram excluídos os casos diagnosticados em pessoas privadas de liberdade e aqueles com endereços inexistentes ou incompletos.

A amostra foi obtida por conveniência, sendo composta por 11 pessoas, delimitada no próprio desenvolvimento do estudo, através do critério de saturação teórica dos dados, que ocorre quando não há novos elementos nas informações coletadas, chegando-se ao adensamento teórico ocasionado pelas repetições^(15)^.

### Coleta e organização dos dados

Para o acesso aos participantes, realizou-se primeiramente um contato com a coordenação de vigilância epidemiológica do município, que forneceu uma lista de pessoas acometidas por TB, cadastradas no Sistema de Informação de Agravos de Notificação (SINAN) entre os anos de 2020 e 2021. A coleta de dados ocorreu nos meses de janeiro e fevereiro de 2024. Ademais, nenhuma pessoa se recusou a participar da pesquisa.

As entrevistas foram realizadas por uma pesquisadora enfermeira, discente de pós-graduação, e por dois alunos de graduação em Enfermagem de uma instituição pública de ensino superior, integrantes do grupo de pesquisa “Avaliação dos Serviços de Saúde” (UEPB/CNPq), que foram treinados pela coordenadora da pesquisa. Os participantes foram abordados pessoalmente, e as entrevistas ocorreram em suas residências.

Para a obtenção dos dados, foi utilizado um roteiro semiestruturado, elaborado pelos pesquisadores, com perguntas sobre as características sociodemográficas e com as seguintes questões norteadoras: “Fale como se deu o seu diagnóstico e tratamento para tuberculose durante a pandemia de COVID-19”. “Fale sobre o acesso aos serviços de saúde para tratamento da TB durante a pandemia”. “Fale sobre a assistência prestada pelos profissionais de saúde durante a pandemia”. O roteiro foi previamente aplicado a uma pessoa que realizou o tratamento para TB, a qual não fez parte da amostra, para avaliar a clareza das questões norteadoras. Os depoimentos foram audiogravados e tiveram duração média de nove minutos. Logo após serem realizadas, as entrevistas foram transcritas na íntegra, havendo devolutiva para aqueles que demonstraram interesse em receber uma cópia, com vistas a possíveis correções.

### Análise dos dados

As respostas dos participantes foram organizadas para compor o corpus textual e, em seguida, processadas pelo software IRaMuTeQ (*Interface de R pour les Analyses Multidimensionnelles de Textes et de Questionnaires*), ferramenta que viabiliza diferentes tipos de análise de dados textuais, dentre elas a Classificação Hierárquica Descendente (CHD), proposta por Reinert^(16)^, que possibilita identificar classes de segmentos de texto, as quais possuem semelhanças no vocabulário entre si e vocabulário diferente dos demais segmentos de texto^(17)^.

Para esta pesquisa, utilizou-se a técnica de Análise de Conteúdo, proposta por Bardin^(18)^, através da categorização temática, contemplando três etapas: pré-análise, na qual foi realizada a leitura flutuante dos depoimentos; seguida pela leitura exaustiva do material, selecionando as unidades de registro. E, por último, as unidades de registro foram organizadas em categorias e interpretadas de acordo com o referencial teórico e a literatura da área. Assim, surgiram quatro categorias temáticas: repercussões da pandemia de COVID-19 no acesso aos serviços de saúde; cuidado às pessoas com tuberculose no contexto pandêmico; implicações da pandemia na busca pelo diagnóstico da TB; implicações da pandemia na adesão ao tratamento da TB.

## RESULTADOS

Dos 11 indivíduos que participaram do estudo, a maioria era do sexo feminino (63,6%), tinha mais de 30 anos (72,7%), considerava-se da cor branca (54,6%), tinha ensino superior completo (45,4%) e possuía trabalho formal (45,4%), com renda entre um e dois salários mínimos (72,8%). No que se refere ao acompanhamento para TB, a maior parte foi acompanhada pela UBS (72,2%) e todos (100%) realizaram o tratamento autoadministrado.

O corpus textual, composto pelas respostas dos participantes sobre o enfrentamento da TB durante a pandemia, abordando o diagnóstico, tratamento, acesso aos serviços de saúde e a assistência prestada pelos profissionais de saúde, apresentou 5.908 ocorrências de palavras e 1.156 formas distintas, com aproveitamento de texto de 81,18%, considerado satisfatório para a análise da CHD.

A análise lexicográfica do corpus, por meio da CHD, agrupou os textos em duas repartições e seis classes, como pode ser visualizado no diagrama que demonstra o dendrograma produzido pela análise do IRaMuTeQ (Figura 1). Nele, também se observam as palavras que tiveram destaque em cada classe, escolhidas entre as que possuíram valor do qui-quadrado (x^2^) igual ou superior a 3,84, seguidas das respectivas porcentagens de segmentos que contêm a palavra nas classes.

**Figura 1:** Diagrama das classes que compõem o dendrograma do corpus textual referente à percepção das pessoas acometidas por tuberculose, Campina Grande, Paraíba, Brasil

*ST- Quantidade de segmento de texto; X2 - qui-quadrado de associação da palavra com a classe; %- porcentagem de segmentos que contêm a palavra na classe.*

Com base nas classes e segmentos de textos evidenciados pela CHD, construíram-se categorias temáticas apoiadas na técnica de Análise de Conteúdo, proposta por Bardin (2016), apresentadas a seguir:

### Categoria I- Repercussões da pandemia de COVID-19 no acesso aos serviços de saúde

A categoria I emergiu da classe 3, que agrupa as percepções dos indivíduos sobre o acesso aos serviços de saúde em meio à pandemia. Destacou-se que o apoio da família foi essencial para que as pessoas com TB evitassem a contaminação pelo Sars-Cov-2 ao acessarem os serviços de saúde para dar continuidade ao tratamento da TB, por exemplo, para retirar as medicações na UBS e levar a coleta de escarro para monitoramento mensal no Ambulatório de Referência em TB.

[...] *ficava em casa e tinha uma pessoa da minha família que, eu fazia o exame do escarro, e ela levava pra lá por conta da pandemia pra que eu não tivesse mais riscos de além da TB pegar uma COVID, essa rede de apoio foi essencial.* (E9)

O acompanhamento da TB realizado pela Atenção Primária à Saúde (APS), que já existia antes da pandemia, foi fundamental para o seguimento do tratamento, visto que, além da proximidade da UBS das residências, os profissionais possuem maior vínculo com a população.

*O acesso aos serviços de saúde durante a pandemia não foi difícil, eu ia pra o posto de saúde perto de casa e os remédios meu pai pegava no posto.* (E3)

*Eu tive todo apoio da família, quando eu precisava, alguém ia buscar as medicações* [...]. (E9)

### Categoria II- Cuidado às pessoas com tuberculose no contexto pandêmico

A segunda categoria surgiu da classe 5, que reuniu falas que caracterizaram a assistência à saúde recebida como sendo boa e acolhedora, conforme observado nos trechos a seguir:

*A assistência prestada pelos profissionais de saúde durante o tratamento para tuberculose foi boa.* (E3)

[...] *o pessoal me tratou bem, foi muito acolhedor.* (E1)

Contudo, as medidas impostas para conter a pandemia, como o distanciamento social, podem ter comprometido a assistência prestada pelos profissionais de saúde, que deixaram de realizar atividades essenciais para o acompanhamento da TB, o que pode ser percebido nos trechos abaixo:

*Pra falar a verdade a assistência prestada pelos profissionais de saúde, eles nem aqui tão vindo, eles não estão vindo me visitar. A UBS é aqui perto da minha casa eles não vêm. Não vieram até agora.* (E4)

*Não tive acompanhamento dos profissionais de saúde, eu imaginei que as pessoas da unidade básica estariam me visitando.* (E9)

A interrupção do tratamento foi relatada por dois entrevistados, favorecendo complicações, como agravamento do quadro clínico.

[...] *a única coisa que aconteceu foi a falta de medicação por uma semana no posto, porque o pessoal estava adoecendo, sendo afastado, então assim teve esse contratempo.* (E10)

*Meu tratamento teve uma quebra, que não era pra ter que a minha preocupação foi essa porque elas perderam minha ficha dentro de uma unidade de saúde.* (E5)

### Categoria III- Implicações da pandemia na busca pelo diagnóstico da tuberculose

A terceira categoria surgiu da classe 2. Na maioria dos relatos, o diagnóstico ocorreu em tempo oportuno. Porém, para alguns entrevistados, decorreu um tempo maior que 30 dias para concluir o diagnóstico. Além disso, tiveram que acessar mais de um serviço, aumentando ainda mais o risco de se contaminar pela COVID-19.

*Já tinha passado bastante tempo já estava com uns 40 dias mais ou menos e eu indo a posto.* (E01)

*Eu fiquei indo ao pneumologista quase que o ano todo, me tratando como um asmático, e foi perdendo tempo, quando foi em 2020, então eu fui pra outro médico, quando ele olhou ele disse vá fazer esse teste de tuberculose.* (E7)

*O ruim era isso que você doente tem que tá passando por locais podendo pegar a COVID se expor mais e mais.* (E8)

Houve também relatos de equívocos e atrasos no diagnóstico. Assim, ocorreram prejuízos no diagnóstico diferencial da TB em relação a outras doenças, como gripe, COVID-19, pneumonia, câncer.

*A médica falou que eu estava com princípio de ansiedade. Só que eu não estava, e eu disse a ela que eu não estava, que eu estava sentindo muitas dores na costela, podia ter tido derrame pleural. Ela disse: não! Que era só uma “gripezinha”, ela passou uma medicação.* (E08)

### Categoria IV- Impacto da pandemia no tratamento para tuberculose

Essa categoria emergiu das classes 4, 1 e 6. Na classe 4, alguns depoimentos enfatizaram o profissional enfermeiro, atuante na UBS, como potencializador para o estabelecimento do vínculo usuário-serviço, sendo capaz de estimular a adesão ao tratamento para TB e alcance da cura, como é observado no trecho a seguir:

*A enfermeira aqui do postinho era muito boa, ela tinha uma espiritualidade muito forte. Eu gostava muito do jeito dela, eu disse pra ela, no final do tratamento, eu agradeci porque ela era de uma autoestima muito grande.* (E01)

Outros entrevistados mencionaram ter recebido orientação para prevenir a transmissão da TB através da separação de objetos pessoais, orientação esta que é cientificamente incorreta e que pode levar a atitudes discriminatórias:

*Minhas coisas era tudo separadas: o prato o copo.* (E10)

*Foi me orientado que com 15 dias, durante 15 dias eu tinha que tá em total isolamento, reservando tudo em casa talheres essas coisas.* (E1)

Na classe 1, foi evidenciado o impacto sofrido pela pessoa acometida por TB em meio a um cenário de incertezas que assolava o mundo, culminando na acentuação das práticas de cuidado para que não pudesse haver complicações graves, conforme demonstrado nas falas a seguir:

*Até da minha família eu fiquei isolada eu moro na parte de cima, então ninguém tinha contato comigo.* (E10)

*O tratamento durante a pandemia foi de muito mais cuidado do que estava sendo* [...] *porque a pandemia veio para assustar.* (E9)

*Primeiramente, veio a maior dificuldade inicial que era o uso da máscara, eu precisava estar em casa de máscara.* (E9)

Para alguns dos entrevistados, o isolamento social imposto pela pandemia foi um fator facilitador para lidar com a doença, no sentido de que foi uma medida imposta à população em geral, contribuindo para uma maior discrição:

*Acabou sendo uma aliada no sentido de que eu não estava sozinha, isolada, não era só eu que precisava me isolar, muitas pessoas, todo mundo teve que ficar em quarentena, em repouso e acabou melhorando.* (E9)

A Classe 6, que também compõe essa categoria, revelou que a pandemia pode ter contribuído para acentuar, também, a estigmatização, como observado através da fala abaixo:

*Isso é contagioso? Com toda essa visão, esse preconceito. O tabu que existe, inclusive, são pessoas que começam a perguntar, aquele preconceito, aquela visão que as pessoas tem que a tuberculose é doença de pobre, é uma doença que está em quem é menos favorecido. Tudo isso. Doença de falta de higiene, e eu não tinha nada disso, minha realidade era outra, ficavam me perguntando: aonde foi que eu peguei isso.* (E9)

Foram realçadas experiências negativas, ocasionadas pelo agravamento da TB, como hospitalização e sequelas, que podem ter sido provocadas pelo atraso na detecção da TB e, consequentemente, pelo início tardio do tratamento, como mostram os seguintes trechos:

*Tinha muita secreção, fiz duas broncoscopias que é uma lavagem pulmonar, ele achou que tinha dado certo, não deu! Ele disse: tem nódulos. Achava que era maligno, mas não foi, e tirou o lóbulo esquerdo inferior, fez um ano.* (E7)

*Passei doze dias internado, dos doze, quatro foi na uti e a médica queria até me entubar só não fiquei entubado porque minha tia não deixou.* (E8)

## DISCUSSÃO

A análise do conteúdo das falas permitiu compreender determinantes ou situações que interferiram, facilitando ou dificultando, de acordo com as dimensões da vulnerabilidade, o diagnóstico, a adesão ao tratamento, o acesso aos serviços e a assistência prestada pelos profissionais de saúde.

As características sociodemográficas da maioria dos participantes não sugerem aspectos de vulnerabilidades individuais, como observado em outro estudo, no qual se apontaram a baixa escolaridade, o uso de álcool e outras drogas, distúrbios psicológicos e a falta de apoio familiar como fatores capazes de interferir na adesão ao tratamento da TB^(19)^.

O papel da família como corresponsável pelo tratamento, apontado na primeira categoria, foi essencial para minimizar os riscos de exposição à contaminação da pessoa com TB pela COVID-19 ao acessar os serviços de saúde. O apoio dos familiares é primordial, pois ajuda a superar as dificuldades que possam surgir e contribui para o sucesso do tratamento^(20,21)^.

Outro fator que pode ter contribuído para que não houvesse implicações no acesso aos serviços durante a pandemia foi a realização do acompanhamento e tratamento da TB na APS, observada na maioria dos relatos, que enfatizaram não ter havido repercussões no acesso, possivelmente devido à proximidade geográfica entre suas residências e a APS^(22)^.

Além disso, o cuidado à pessoa com TB, abordado na segunda categoria, requer uma assistência voltada à promoção do vínculo com as equipes e os serviços de saúde, sendo de extrema importância no enfrentamento da doença, pois permite a identificação das necessidades individuais e a compreensão das vulnerabilidades que possam influenciar o desfecho, o que possibilita o desenvolvimento de cuidados específicos, com a participação de outros serviços^(23)^.

Nesse sentido, o trabalho das equipes de saúde deve ser guiado por ações que favoreçam a adesão ao tratamento, tais como: acolhimento, escuta ampliada, visita domiciliar (VD), tratamento diretamente observado (TDO), ações de educação em saúde que promovam a superação do estigma e da discriminação, entre outras^(10)^.

O contexto pandêmico, com as necessárias orientações para conter a disseminação da COVID-19, como o distanciamento social, pode ter comprometido a realização de atividades de controle e acompanhamento da TB^(10)^, enfatizadas pelos indivíduos do presente estudo, e que configuram vulnerabilidades programáticas, as quais refletem deficiências nos sistemas de saúde^(23)^, como a não realização de visitas domiciliares e do TDO, já que todos realizaram o tratamento de forma autoadministrada.

A VD é uma das recomendações do Ministério da Saúde para que os serviços fortaleçam o vínculo e a manutenção do tratamento. Desse modo, recomenda-se a VD, sempre que possível, a todos os pacientes, principalmente no início do tratamento, possibilitando a compreensão do contexto social em que estão inseridos^(3)^.

O TDO também constitui uma ferramenta fundamental, humanizada e comprometida para o monitoramento do tratamento. Sua realização permite, ainda mais, a aproximação dos profissionais com o contexto social dos indivíduos^(23)^. Sendo assim, o TDO destina-se a todas as pessoas com diagnóstico de TB e consiste na supervisão da tomada dos medicamentos de três a cinco vezes por semana, visando diminuir a interrupção do tratamento medicamentoso^(3)^.

A pandemia de COVID-19 acentuou os desafios, mas também evidenciou fragilidades que já não deveriam existir, principalmente na APS, pois o tratamento da TB é prioritário, de acordo com a Política de Atenção Básica^(6)^.

Nessa perspectiva, pode-se citar que o Plano Nacional pelo Fim da TB apoia o desenvolvimento de estratégias que utilizem, por exemplo, tecnologias digitais^(2,10)^, assim como as próprias orientações internacionais e nacionais para o manejo e controle da TB durante a pandemia reafirmaram que eram necessários esforços para o fortalecimento do vínculo com as pessoas em tratamento para TB, mesmo à distância, por meio de chamadas telefônicas ou videochamadas, que, no entanto, não foram relatadas pelos entrevistados, inferindo-se que essa organização não ocorreu^(7,24)^.

Em um estudo realizado no estado da Paraíba, constatou-se que não houve subsídio para a concretização das ligações telefônicas, e que a maioria das UBS não possuía aparelhos telefônicos ou celulares. Dessa forma, atividades que eram rotineiras da APS ficaram paralisadas durante a pandemia, fato que suscita a necessidade de maiores investimentos que garantam a realização das ações de controle para TB e outras doenças prioritárias, mesmo em períodos adversos^(25)^.

A interrupção do tratamento para TB relatada também demonstra que a pandemia provocou um aumento da perda de seguimento do tratamento, com a desestruturação ou desorganização dos serviços, devido à realocação de recursos materiais e humanos e à própria recomendação de redução da frequência dos atendimentos em função da COVID-19, enfatizando a vulnerabilidade programática^(26)^.

Esses aspectos de vulnerabilidade também foram observados em um estudo de coorte retrospectiva realizado no Brasil, nos anos de 2020 e 2021, o qual mostrou que a proporção de perda de seguimento do tratamento aumentou em decorrência da pandemia, alcançando aproximadamente 19%, valor três vezes maior do que o recomendado pela OMS^(3,26)^.

Em relação ao diagnóstico da TB em meio à pandemia de COVID-19, na terceira categoria, constatou-se o atraso de mais de quarenta dias em um caso e de um ano em outro para o diagnóstico da TB, embora a maioria dos entrevistados tenha sido diagnosticada em tempo oportuno. Houve também erros no diagnóstico diferencial da TB, que contribuem ainda mais para a disseminação da doença na sociedade e sugerem vulnerabilidades programáticas.

As pessoas com diagnóstico de TB, devido ao comprometimento pulmonar, fazem parte do grupo de risco para a COVID-19 e podem apresentar sintomas semelhantes entre as duas doenças, como febre e tosse. Por esse motivo, precisam ser vistas com atenção especial, voltada ao diagnóstico diferencial e à detecção precoce^(27)^.

O atraso no diagnóstico possivelmente está relacionado à influência da pandemia, que culminou na queda do diagnóstico de TB em todo o mundo. De acordo com o Boletim Epidemiológico^(11)^, na Paraíba, no ano de 2020, foram notificados 1.144 novos casos, com uma redução na detecção em comparação ao ano anterior, demonstrando que os serviços não conseguiram manter as ações de vigilância e controle da TB durante a pandemia^(28)^, o que pode favorecer a disseminação da doença na sociedade. Por isso, o diagnóstico precoce e o início imediato do tratamento são importantes ações de controle da TB^(4)^.

Na quarta categoria, que aborda o impacto da pandemia no tratamento da TB, houve o relato de três participantes (E4, E9 e E7) a respeito do agravamento dos sintomas, com necessidade de hospitalização. Dentre eles, o E7, devido ao atraso no diagnóstico, precisou realizar uma lobectomia pulmonar (retirada do lóbulo esquerdo inferior) e hoje convive com essa sequela. Esses dados confirmam que, mesmo sendo uma doença curável, vulnerabilidades programáticas podem levar a um prognóstico desfavorável, e remetem à necessidade de maior preparo dos profissionais para a detecção e diagnóstico precoce da TB^(23)^.

Ainda nessa categoria, foi enfatizado o apoio dos profissionais, com destaque para o profissional enfermeiro. Outras pesquisas mostram que a enfermagem é protagonista no enfrentamento e controle da doença, principalmente na APS, que permite uma relação mútua entre profissional e usuário e também o reconhecimento das necessidades de saúde. É através dessa relação que surgem a confiança e a autonomia, que ajudam as pessoas acometidas pela TB a superarem medos, receios e estigmas em relação à doença e ao tratamento^(25,29)^.

No entanto, é importante destacar que a orientação incorreta sobre a transmissão da TB, recomendando a separação de talheres, copos e pratos, revela vulnerabilidade programática. Sabe-se que a transmissão do *Mycobacterium tuberculosis* ocorre por via aérea, a partir da eliminação dos bacilos por meio da fala, tosse ou espirro, que ficam suspensos no ar^(4)^.

Assim, o compartilhamento de informações separatistas como essa não pode partir de profissionais de saúde, que devem orientar os usuários desde a suspeição da doença até a obtenção da cura, com informações essenciais, como a definição da TB, formas de prevenção, a importância do controle dos comunicantes, o tempo de tratamento, as reações adversas e a necessidade de aderir ao tratamento para alcançar a cura^(20,27)^. Desse modo, é necessário que haja qualificação desses profissionais, com educação continuada, para atuar no acompanhamento do doente de TB.

Além do enfrentamento da TB, era necessário lidar também com a pandemia de COVID-19, acentuando a obrigatoriedade de medidas de etiqueta respiratória, higienização das mãos e uso de máscaras faciais^(30)^ para conter a disseminação do novo coronavírus e proteger-se de uma possível coinfecção. Esses fatores podem ter se configurado como mais um desafio na vida de quem já estava tratando uma grave doença pulmonar, como a TB. Um dos entrevistados relatou que o uso de máscara era um desafio, devido à dificuldade de respirar.

Embora essas medidas tenham causado mudanças nos aspectos comportamentais e emocionais das pessoas, para o enfrentamento da TB, o isolamento social foi visto por um dos entrevistados como um aliado, pelo fato de que todos estavam em quarentena. Assim, não foi preciso dar explicações sobre a doença, o que possibilitou evitar o preconceito que ainda assola o enfrentamento da TB.

Concepções preconceituosas sobre a TB persistem nos dias atuais, mesmo sendo uma doença curável, e acentuam a estigmatização. Como relatado por um entrevistado, o adoecimento por TB ainda é associado a comportamentos desregrados e falta de higiene. O preconceito surge, muitas vezes, de crenças equivocadas sobre o contágio da doença, que povoam o imaginário social, contribuindo para a vulnerabilidade social^(6,20)^. Nesse sentido, é necessário promover espaços de discussão com a população sobre a TB, principalmente no que se refere à sua transmissão e como evitá-la, para que mudanças nessas concepções possam ocorrer, desmistificando e conscientizando todos. Dessa forma, a pessoa acometida por TB pode se sentir acolhida e reinserida na sociedade.

### Limitações do estudo

Destacam-se, como limitações do estudo, a dificuldade de acesso ao banco de dados do SINAN, o tamanho reduzido da amostra e a incompletude dos dados e/ou preenchimento incorreto, principalmente no que se refere aos endereços, o que dificultou a realização das entrevistas.

### Contribuições para a área de Saúde Pública

Este estudo possibilita compreender, sob a perspectiva das pessoas com tuberculose, como ocorreu o tratamento da doença durante a pandemia de COVID-19, o que evidencia a necessidade de fortalecimento de estratégias que garantam o enfrentamento de doenças negligenciadas, como a tuberculose, no contexto de uma emergência de saúde pública.

## CONSIDERAÇÕES FINAIS

Os achados permitiram compreender que a pandemia contribuiu para gerar ou potencializar vulnerabilidades programáticas e sociais, que impactaram o enfrentamento da TB, como: a não realização das visitas domiciliares e do TDO, a interrupção do tratamento, o atraso e equívocos no diagnóstico, além da estigmatização.

Em contrapartida, o apoio da família, o acompanhamento do tratamento da TB pela APS, a assistência acolhedora, o papel do enfermeiro como potencializador da adesão ao tratamento e o próprio isolamento social, que ajudou a reduzir o preconceito, foram essenciais para o alcance da cura da TB em meio ao cenário pandêmico adverso.

Fica evidente a relevância e a urgência da ampliação das ações de controle e acompanhamento da TB, para que seja possível alcançar os ODS e eliminar a TB como um problema de saúde pública. Dessa forma, acredita-se que é necessário promover educação permanente em saúde sobre o manejo e controle da doença, de forma efetiva, para os profissionais de saúde; proporcionar educação em saúde para a população, para que todos possam se engajar na luta contra a TB; e fortalecer políticas e investimentos para que as ações possam ser mantidas, mesmo em períodos de crises sanitárias.
